# Sanitary Matters in the West African Colonies

**Published:** 1909-08-28

**Authors:** 


### Sanitary Matters in the West African Colonies.

Professor Simpson, after visiting the Gold Coast to investigate the recent outbreak of plague there, studied the sanitary conditions prevailing in some of the other West African colonies. His results are embodied in a report which is full of information and instruction. He found the towns in West Africa terribly behind the times in matters relating to sanitation, because at present the West African Medical Staff is not equipped for dealing with practical sanitation, and no one appears to be responsible for the public health. From time to time individual medical men and the Government attempt to improve matters, but owing to the fact that few officers are permanently appointed to one station there is no definite continuity in any policy. Further, most of the men have their ordinary work to perform, and it is only in their off times that they can take an interest in destroying mosquitoes, looking at drains, the construction of houses and so on. Nowadays it is realised that no great advance in public health can be made without a special health department, and the remedy for the unhealthy condition of the West Coast towns is to be found, as Professor Simpson suggests, in the creation of a special Health Organisation.

"It is impossible," he remarks, "to effect permanent and important improvements, and when effected to maintain them, without an organisation whose duty is to initiate and advise the Government as to improvements, and when these have been sanctioned, to see that they are properly carried out. Without an organisation no proper inspection of out-stations and native towns can be systematically carried out; recommendations have not the weight they would otherwise possess, and money is wasted, full value being seldom obtained for the expenditure. Owing to absence of a special sanitary administration drains are constructed that are useless; houses are built that are unhealthy; towns are allowed to grow up from villages without any forethought as to development and growth, the result being often a most insanitary condition that nothing but costly demolition will remedy or remove. Places become or continue to be malarious, because there is no organisation or controlling agent to prevent it. Moreover, an organisation such as this will be able to deal with important problems. Apart from the prevalence of malaria and tuberculosis, other diseases such as yellow fever, sleeping sickness, smallpox, cerebro-spinal disease, and plague, have to be carefully watched, and measures taken to prevent their spread both by land and sea. Besides this inter-provincial and inter-colonial risk of infection there are risks arising from the increasing trade of West Africa with European ports. Cholera is a disease from which West Africa has hitherto been free, and owing to the bad water supply in the country generally it is of the highest importance that every precaution shall be taken to prevent the introduction of a disease which would be more destructive than plague."

Professor Simpson proposes that there shall be a special health department in each colony, small in its constituent numbers, whose members should be specially trained, and whose whole time should be devoted to public health duties. This department should be a branch of the West African Medical Staff separate in its functions and not transferable to other medical duties, and under a Sanitary Commissioner. It would be advisable, in order to prevent friction from a dual system of control, for the Sanitary Commissioner to be subordinate to the principal medical officer as President of the Central Board of Health, but yet in sanitary matters to be recognised as the expert, and as the responsible officer. The executive control of the health organisation should be completely under the Sanitary Commissioner. No medical officer of the West African Medical Staff should be eligible to enter the special health branch or department until he has been four or five years in the colony as an ordinary medical officer, and no officer should hold a public health appointment unless he holds or takes within a certain time of his appointment a diploma in public health and a diploma in tropical medicine and hygiene. When once in the department, promotion should be in that branch up to the Sanitary Commissioner, and not in the medical branch leading to the principal medical officership.

This, briefly, is the scheme, which seems well worthy of consideration. An obstacle in its way will probably be the question of funds to finance it. It is manifest, however, that ultimate improvement in all these West African towns will lower the death rate and so save the Government money in the long run on the lives of their employés; ultimately some such scheme must find a place in the future development of West Africa.

